# A CBCT evaluation of molar uprighting by conventional versus microimplant-assisted methods: an in-vivo study

**DOI:** 10.1590/2177-6709.23.3.35.e1-9.onl

**Published:** 2018

**Authors:** Sergio Martires, Nandini V. Kamat, Sapna Raut Dessai

**Affiliations:** 1 Goa Dental College and Hospital, Department of Orthodontics and Dentofacial Orthopedics (Bambolim, India).; 2 Goa Dental College and Hospital, Department of Oral Medicine, Diagnosis and Radiology (Bambolim, India).

**Keywords:** Dental implant, Molar, Cone-beam computed tomography

## Abstract

**Objective:**

The aim of this prospective study was to compare the three-dimensional effects of the conventional helical uprighting spring (CA) and the mini-implant assisted helical uprighting spring (MIA), using CBCT scans.

**Methods:**

Twenty patients with mesially tipped second mandibular molars were divided into two groups: CA group, in which 10 patients were treated using a conventional helical uprighting spring with conventional anchorage; and MIA group, in which 10 patients were treated using a mini-implant supported uprighting spring. Molar uprighting was observed in both groups for a period of four months. Two standardized 11×5-cm CBCT sections of the mandible were taken, being one prior to uprighting and one at the end of the four month follow-up. Statistical analyses at the beginning of treatment and after a 4 month follow-up were performed, with a significance level of *p*< 0.05.

**Results:**

The mean amount of change in mesiodistal angulation in the MIA group was 8.53 ± 2.13^o^ (*p*< 0.001) and in the CA group was 9.8 ± 0.5^o^ (*p*< 0 .001). Statistically significant differences were found between the two groups with regard to buccolingual inclination of canine, first and second premolars (*p*< 0.05), second molar (*p*< 0.001) and extrusion of second molar (*p*< 0.05).

**Conclusions:**

The mean amount of change in the mesial angulation of the second molar in the CA as well as the MIA groups was similar. MIA, which used mini-implant as a source of anchorage, was more effective in preventing movement of the anchorage teeth as well as preventing extrusion of the second molar in the vertical plane, when compared to the CA group, which used dental units as a source of anchorage.

## RESUMO

**Objetivo:**

o objetivo deste estudo prospectivo foi comparar, usando imagens de TCFC, os efeitos tridimensionais da mola convencional de verticalização (CA) e da mola de verticalização com ancoragem em mini-implantes (MIA).

**Métodos:**

vinte pacientes com segundos molares inferiores inclinados mesialmente foram divididos em dois grupos: grupo CA, no qual 10 pacientes foram tratados usando mola helicoidal de verticalização com ancoragem convencional; e grupo MIA, com 10 pacientes tratados usando mola de verticalização ancorada em mini-implantes. A verticalização dos molares foi observada nos dois grupos por um período de quatro meses. Foram obtidas duas secções tomográficas da mandíbula, com dimensões padronizadas de 11 x 5 cm, uma antes da verticalização e outra ao fim dos quatro meses de acompanhamento. As análises estatísticas ao início do tratamento e após os quatro meses de acompanhamento foram realizadas a um nível de significância de *p*< 0,05.

**Resultados:**

a média das alterações na angulação mesiodistal do grupo MIA foi de 8,53 ± 2,13^o^ (*p*< 0,001), e do grupo CA foi de 9,8 ± 0,5^o^ (*p*< 0,001). Diferenças estatisticamente significativas foram encontradas entre os dois grupos em relação à inclinação vestibulolingual do canino, do primeiro e segundo pré-molares (*p*< 0,05) e do segundo molar (*p*< 0,001), bem como para a extrusão do segundo molar (*p*< 0,05).

**Conclusões:**

a alteração média na angulação mesial do segundo molar nos grupos CA e MIA foi semelhante. O método MIA, que usou mini-implantes como ancoragem, foi mais efetivo na prevenção da movimentação dos dentes de ancoragem, bem como na prevenção da extrusão do segundo molar no plano vertical, quando comparado com o grupo CA, em que dentes foram usados como fonte de ancoragem.

## INTRODUCTION

Permanent first molars are the first permanent teeth to erupt into the oral cavity. Due to their presence for a longer time in the oral cavity, they are highly prone to caries and are generally lost early with the lack of proper oral hygiene. This results in inclination and rotation of the second and sometimes third molars, associated with periodontal problems, distal movement of the canine and premolars and extrusion of the antagonist first molar.^1^

Loss of a permanent first molar should be addressed immediately by prosthetic replacement or orthodontic space closure, to avoid functional and anatomical disturbances.^1^ Preparation of a tipped tooth for a fixed prosthesis necessitates excessive reduction on the mesial aspect to produce an acceptable path of insertion, which may lead to pulp exposure.^2^ To prevent this, molar uprighting could be carried out in order to help with the development of an optimal periodontal environment.

Sawicka et al^3^ have demonstrated how the conventional helical uprighting spring used for molar uprighting produces effects on the tooth in three planes of space. It is not possible to assess effects in all three planes of space with conventional 2D radiographs and the buccolingual dimension is rendered inaccessible. In such cases, the CBCT imaging modality could be used as reliable tool to assess the effects in all three planes of space.

The objective of the present clinical study was to compare the effects of the conventional uprighting spring and of the mini-implant assisted molar uprighting spring, using the 3D CBCT scans. 

## MATERIAL AND METHODS

A study was conducted on 20 patients with a mean age of 26.9 years in the Department of Orthodontics and Dentofacial Orthopedics in the Goa Dental College and Hospital. It was approved by a scientific ethical committee, according to Ref. No. Ethical Comm./GDCH/2015-2/Ortho-1 and an informed consent was obtained from all the patients.

Healthy patients with missing first molars and mesially tipped second molars with a healthy periodontium were included in the study, while patients with untreated systemic conditions and loss of periodontal attachment were excluded.

**Figure 1:**
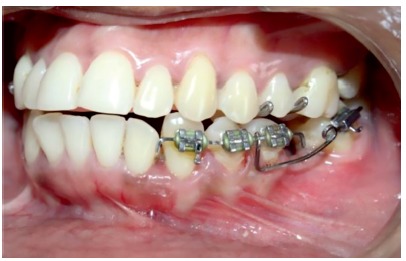
Helical uprighting spring with conventional anchorage unit.

» All twenty patients were randomly divided into two groups. Randomization was done using lottery method.
» CA group: consisted of 10 patients (6 females and 4 males) with a mean age of 28.7 years who were treated by using a conventional helical uprighting spring using canine, first and second premolars as anchorage teeth.
» MIA group: consisted of 10 patients (7 females and 3 males) with a mean age of 25.1 years who were treated using a mini-implant supported uprighting spring, where the mini-implant was placed inter-radicularly between the first and second premolars
» For the patients in the CA group, brackets were placed on the canine, first and second premolars as per convenience position, to allow the passive placement of a 0.019 ×0.025-in SS wire and a single 0.022 ×0.028-in tube was placed on the second molar, which was to be uprighted. The entire assembly consisted of the second molar to be uprighted and the anchorage unit which was comprised of the canine, first and second premolars. An uprighting spring (15-20 mm in length) made with 0.017 × 0.025-in SS wire (3M Unitek, Monrovia, CA, USA) was fabricated, passing through the molar tube, left uncinched and hooked onto the anchorage unit wire anteriorly, between the brackets of the first and second premolars (Fig 1). The spring was given a lingual bend before hooking it to the anchorage unit.

**Figure 2:**
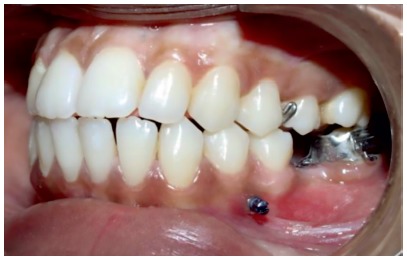
Mini-implant placed interradicularly between the two mandibular premolars.

**Figure 3:**
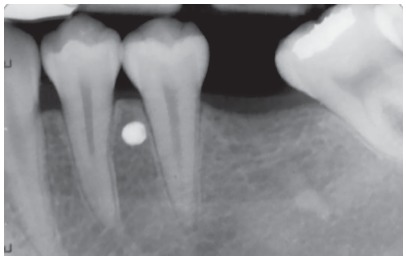
Periapical radiograph after mini-implant placement.

**Figure 4:**
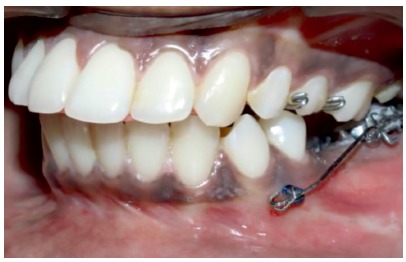
Uprighting spring attached to the head of the mini-implant.

» For the patients in the MIA group, a 0.022 × 0.028-in tube was placed on the second molar, which was to be uprighted. A self-drilling mini-implant (S.K. Surgicals, Pune/India), 1.5 mm in diameter and 8 mm in length, was placed interradicularly between the first and second premolars (Fig 2) and intraoral periapical radiographs (IOPAs) were used to confirm its position (Fig 3). An 0.017 × 0.025-in SS (3M Unitek, Monrovia, CA USA) uprighting spring was fabricated to pass through the molar tube, left uncinched and hooked directly onto the mini-implant anteriorly (Fig 4). 

**Figure 5:**
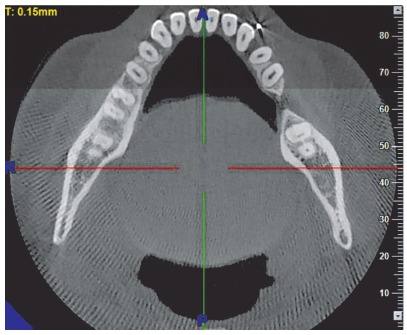
11 × 5-cm CBCT section of the mandible.

**Figure 6:**
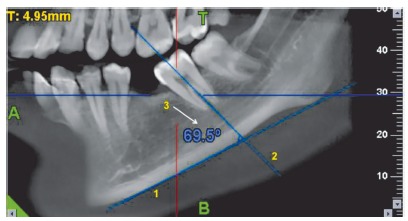
Calculation of mesiodistal angulation of the second molar on an 11×5-cm CBCT section of the mandible.

» The uprighting force was assessed by using a Dontrix gauge (Leone Orthodontics, Italy) to be about 50 g, checked at the level of the wire passing through the anchorage unit for the CA group and at the level of the mini-implant for MIA group. An anterior bite plate was delivered to provide posterior disocclusion and prevent occlusal trauma. At four weeks intervals, the force level was checked and reactivated if required.
» Molar uprighting was observed for both groups for a period of four months, using two standardized CBCT scans of the mandible - with 11×5-cm field of view (FOV) and 0.15-mm^3^ *voxel* dimensions -, one prior to uprighting and one at the end of the four month follow-up (Fig 5). The CBCT equipment utilized was NewTom Giano (Cefla Dental, Italy) and was operated at 90 kV and 3 mA. Various parameters were assessed using the NNT Viewer v. 5.1 software. The CBCT sections were obtained for all patients by positioning them in the natural head position (NHP). This was done in order to maintain standardization of all patients. The literature^4^ suggests that the reproducibility of NHP is close to 2^o^ on repeated radiographs. NHP is a reproducible position when used for bidimensional radiography, the subject is expected to exhibit same reproducibility in position when taking 3D scans. To measure the mesiodistal angulation (Fig 6), the required sagittal sections were obtained by turning the scanned volume in such a way that the desired sagittal plane was obtained by passing through the central grooves of the posterior teeth of the desired side as well as the canine tip. When this was done, the sagittal slice thickness was increased to visualize both the angle and the lower border of the mandible. A line was then drawn to the lower border of the mandible.^1^ A line passing through the long axis of the molar was drawn to contact the tangent to the lower border.^2^ The inner angle was then used to measure the mesiodistal angulations.^3^ These measurements were carried out on the sagittal sections.

**Figure 7:**
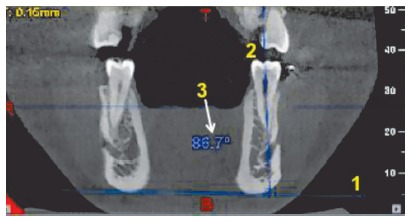
Calculation of buccolingual inclination of the teeth, on an 11×5 -cm CBCT section of the mandible.

» After locating the desired tooth on the axial image for assessment of buccolingual inclination (Fig 7) separately for molar, premolars and canine, the sagittal section for the same tooth was observed and oriented in such a manner that the complete length of the desired tooth was seen in the coronal plane. A line was traced by marking the lowermost points on the inferior border of the mandible bilaterally^1^ in the coronal section along with a line passing through the central groove and root apex of the molars and premolars and through the cusp tip and root apex of the canine.^2^ The inner angle was used to obtain the buccolingual inclination of the tooth in question.^3^ The planes were standardized and showed reproducibility. Similar measurements were then made to evaluate the bucco-lingual inclinations of the canine, first and second premolars.

**Figure 8:**
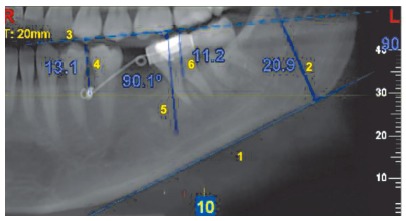
Calculation of the extrusion of the second molar in the vertical plane.

» To calculate molar extrusion in the vertical plane (Fig 8), a tangent was drawn to the lower border of the mandible.^1^ A standard occlusal plane was used to measure the extrusive movement of the molar. To determine the posterior limit of the standard occlusal plane, a perpendicular line was drawn to the first point of contact of the tangent to the lower border of the mandible, and a fixed vertical height was considered in the pre and after four month follow-up.^2^ Anteriorly, the most prominent incisor was considered. To confirm the reliability of the plane,^3^ the distance from the plane to the center of the mini-implant on both pre and post scans was calculated.^4^ As these measurements were standardized, the plane was used for extrusive measurements. The center of resistance was marked for the second molar at the level of the furcation, a perpendicular reference line was drawn^5^ and the vertical distance from the center of resistance of the molar to the constructed plane was calculated.^6^ These measurements were also carried out on the sagittal sections.

**Figure 9:**
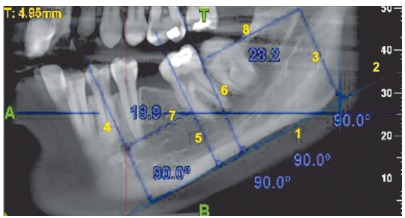
Assessment of the type of uprighting.

» The type of uprighting movement (Fig 9) was then assessed by drawing a tangent to the lower border of the mandible. This was considered to be the x-coordinate.^1^ A perpendicular to the tangent was drawn passing through the constructed gonion^2^ ^,^ ^3^ and another perpendicular to the tangent was constructed passing through the mental foramen.^4^ These two perpendicular lines were considered to be the y-coordinate. The distance from the distal height of the molar crown contour to the posterior perpendicular passing through constructed gonion,^6^ ^,^ ^8^ and the distance from the junction of the apical and middle third of the mesial root to the anterior perpendicular^5^ ^,^ ^7^ passing through the mental foramen were measured. These distances were used to determine if the uprighting had occurred by more of mesial root movement or distal crown movement.

### Statistical analysis

The descriptive statistics were represented as mean and standard deviations for all the assessed parameters. In each group, normality of the data was assessed with Kolmogorov-Smirnov and Shapiro-Wilks tests. Statistical homogeneity of variance was checked using Levene’s test. The independent *t*-tests were used to evaluate the intergroup differences of the degree of change in the initial two parameters and the millimetric change in the remaining parameters. Paired *t*-test was used to evaluate the change between the pre-treatment and the post four month follow-up measurements. An error test was performed using Dahlberg’s formula^5^ and the exact error was described in millimeters or degrees.

## RESULTS

Molar uprighting was carried out for a period of four months in both groups. The amount of change in mesiodistal angulation, change in buccolingual inclination and degree of molar extrusion were calculated using 11×5-cm CBCT sections of the mandible. T_1_ and T_2_ values are given in Table 1.

**Table:** 

Parameter	Conventional anchorage			Mini-implant anchorage			P value (difference of both groups)		
	T_1_ (mean+SD)	T_2_ (mean+SD)	T_2_-T_1_	P value	T_1_ (mean+SD)	T_2_ (mean+SD)	T_2_-T_1_	P value	
Mesiodistal angulation	76.4 ± 6.96	86.2 ± 7.46	9.8 ± 0.5	0.000	79.6 ± 7.89	88.13 ± 5.76	8.53 ± 2.13	0.000	0.499
Buccolingual inclination of canine	97.6 ± 6.18	98.95 ± 5.34	1.35 ± 0.84	0.086	100.27 ± 4.3	99.9 ± 4.78	-0.37 ± 0.48	0.694	0.004
Buccolingual inclination of 1^st^ premolar	91.21 ± 3.97	95.45 ± 3.18	4.24 ± 0.79	0.000	91.63 ± 4.44	92.41 ± 4.46	0.78 ± 0.02	0.259	0.003
Buccolingual inclination of 2^nd^ premolar	85.04 ± 3.92	88.17 ± 4.48	3.13 ± 0.56	0.002	82.95 ± 3.64	83.54 ± 3.03	0.59 ± 0.61	0.435	0.029
Buccolingual inclination of 2^nd^ molar	67.45 ± 5.42	72.01 ± 5.92	4.56 ± 0.5	0.022	65.82 ± 4.09	65.69 ± 4.75	-0.13 ± 0.66	0.935	0.000
Extrusion of molar in the vertical plane	12.56 ± 1.63	12.160 ± 1.8	-0.4 ± 0.17	0.038	9.77 ± 1.76	9.74 ± 1.79	-0.03 ± 0.03	0.193	0.008
Bone density in HU at the mesial alveolar crest	745.80 ± 328.722	1105.30 ± 459.75	359.5 ± 131.032	0.018	988.90 ± 529.46	1208.20 ± 459.873	219.3 ± 69.59	0.027	0.623

The mean amount of change in mesiodistal angulation in the MIA group was 8.53 ± 2.13^o^ and in the CA group it was 9.8 ± 0.5^o^. The difference in the amount of change in mesiodistal angulation between the MIA and the CA groups was found to be statistically insignificant (*p*> 0.05).

The mean amount of change in the buccolingual inclination of the canine in the MIA group was- 0.37 ± 0.48^o^ and in the CA group it was 1.35 ± 0.84^o^. 

The mean amount of change in the buccolingual inclination of the first premolar in the MIA group was 0.78 ± 0.02^o^ and in the CA group it was 4.24 ± 0.79^o^.

The mean amount of change in the buccolingual inclination of the second premolar in the MIA group was 0.59 ± 0.61^o^ and in the CA group it was 3.13 ± 0.56^o^. The differences in the amount of change in the buccolingual inclination of the canine, first and second premolars between the MIA and the CA groups were found to be statistically significant (*p*< 0.05).

The mean amount of change in the buccolingual inclination of the second molar in the MIA group was -0.13 ± 0.66^o^ and in the CA group it was 4.56 ± 0.5^o^. The difference in the amount of change in the buccolingual inclination of the second molar between the MIA and the CA groups was found to be highly statistically significant (*p*< 0.001).

The mean amount of extrusion of the second molar in the MIA group was -0.03 ± 0.03 mm and in the CA group it was -0.4 ± 0.17 mm. The difference in the amount of molar extrusion between the MIA and CA groups was found to be statistically significant (*p*< 0.05).

The mean amount of molar uprighting which took place by root movement was 0.64 mm and the mean amount of movement which took place by distal crown tipping was 0.14 mm in the MIA group. 

The mean amount of molar uprighting which took place by root movement was 0.24 mm and the mean amount of movement which took place by distal crown tipping was 1.03 mm, in the CA group. The mean difference of the two types of molar uprighting movements was 0.5 mm and -0.79 mm in the MIA and CA groups, respectively and were statistically significant (*p*< 0.05) (Table 2).

**Table 2:** Mean difference of the two types of molar uprighting movements for the mini-implant anchorage (MIA) and conventional anchorage (CA) groups.

Group	Mesial root movement T_1_	Mesial root movement T_2_	Mean difference for mesial root movement	Distal crown tipping T_1_	Distal crown tipping T_2_	Mean difference for distal crown tipping	P value
MIA group	20.77	20.13	0.64	28.34	28.2	0.14	0.041
CA group	19.96	19.72	0.24	27.1	26.07	1.03	0.019

The error test done according to Dahlberg’s formula^5^ (Table 3).

**Table 3:** Error test for which parameter (in degrees) for mesiodistal angulation and buccolingual inclination (limit <1.5°) and extrusion of second molar (limit < 1mm).

Parameter	CA Group	MIA Group
Mesiodistal angulation of second molar	o.6	0.4
Buccolingual inclination of canine	0.6	0.5
Buccolingual inclination of first premolar	0.5	0.4
Buccolingual inclination of second premolar	0.6	0.5
Buccolingual inclination of second molar	0.7	0.6
Extrusion of molar in the vertical plane	0.3	0.3

## DISCUSSION

In the present prospective study, 20 patients who presented with mesially tipped second mandibular molar and sound periodontium were divided into two groups: one of conventional helical uprighting spring (CA group) and the other, mini-implant assisted molar uprighting (MIA group). Both groups were evaluated for a period of four months.

Different authors have pointed out that a significant amount of molar extrusion as well as a significant movement of the anchorage unit occurs with most conventional molar uprighting appliances.^3^ ^,^ ^6^ ^-^ ^11^

The advent of mini-implants has brought about a new chapter in orthodontics. The use of mini-implants for molar uprighting has been delineated by different authors^12^ ^,^ ^13^ ^,^ ^14^ who have shown that when using mini-implants there are no side effects on the anterior teeth. Miyahira et al^15^ and Nienkemper et al^16^ have pointed out how miniscrews could be used to alter mechanics and produce molar intrusion during uprighting. Most of these papers were case reports and two of them, review articles. 

Although FEM studies^10^ ^,^ ^17^ ^,^ ^18^ on molar uprighting are available, they do not accurately simulate the clinical situation. Since no study was available in literature so far, comparing the effect of conventional uprighting spring and mini-implant assisted molar uprighting, the present study was carried out. More importantly, in this study the three-dimensional CBCT modality was utilized for the assessment of molar uprighting which also was unprecedented. As such the CBCT imaging modality permitted an accurate analysis of the effects of the two appliances used for molar uprighting in all three planes of space which would otherwise be rendered impossible using 2D imaging modalities.

The radiation dose of 0.078µSv was still adequate according to the recommended annual effective dose limit as stated by the National Council on Radiation Protection and Measurements (NCRP, 2004).^19^

Besides, the CBCT machine used in the present study was the NewTom^®^, which used a lower radiation dose when compared to other CBCT machines such as CB Mercuray and i-CAT.^20^ For this study, the length of the springs was in the range of 15 to 20 mm. However it was not possible to standardize the length of the springs as the degree of tipping of the second molar and the amount of space remaining of the missing first molar varied from patient to patient.

### Amount of change in mesiodistal angulation of the second molar

The mean amount of change in mesiodistal angulation in the MIA group was 8.53 ± 2.13^o^ and in the CA group it was 9.8 ± 0.5^o^. Although the amount of change in mesiodistal angulation was higher in the CA group as compared to the MIA group, the difference between the two groups was not found to be statistically significant.

Kumar et al^21^ evaluated the changes in mesiodistal angulation using lateral cephalograms, and found that the mean amount of change in the mesiodistal angulation was 11.2^o^, which was found to be statistically significant. However their study evaluated only the simple technique of molar uprighting and did not compared it with any other technique. Also, the follow-up period was two months, while in the present study a follow-up of four months was carried out.

### Amount of change in the buccolingual inclination of the canine, first and second premolars

In the present study, the mean amount of change in the buccolingual inclination was carried out on each of the anchorage teeth: canines, first and second premolars, and second molars. The mean amount of change in the buccolingual inclination of the canine in the MIA group was -0.37 ± 0.48^o^ and in the CA group it was 1.35 ± 0.84^o^. This showed that in the MIA group, the canine minimally moved to lingual, whereas in the CA group, the canine moved buccally to a slightly greater degree. 

The mean amount of change in the buccolingual inclination of the first premolar in the MIA group was 0.78 ± 0.02^o^ and in the CA group it was 4.24 ± 0.79^o^. This showed that in the MIA group, the first premolar moved minimally to the buccal, whereas in the CA group, the first premolar moved significantly to the buccal. 

The mean amount of change in the buccolingual inclination of the second premolar in the MIA group was 0.59 ± 0.61^o^, and in the CA group it was 3.13 ± 0.56^o^. this showed that in the MIA group, the second premolar moved minimally to the buccal, whereas in the CA group, the second premolar moved significantly to the buccal. The difference in the amount of change in the buccolingual inclination of the canine, first and second premolars between the two groups was found to be statistically significant(*p*< 0.05).

Kojima et al^10^ conducted an FEM study with a conventional helical uprighting spring and found that in the anchorage teeth, the position of the intrusive force was located away from the center of resistance to the buccal side and therefore a moment tending to produce buccal crown movement was produced. However when the spring arm was bent towards the lingual side before activation, the initial activation produced a force tending to move the anchorage teeth towards the lingual. This force produced a moment in the opposite direction of that produced by the intrusive force, thus cancelling each other out. In the present study, the spring arm was bent toward the lingual before activation. However it was seen that, in the CA group, most anchorage teeth moved significantly towards the buccal, suggesting that the intrusive force causing buccal movement was stronger than the lingual movement caused by the bending of the spring arm, resulting in a net buccal movement. In the MIA group, minimal movement of the anchorage teeth was seen.

### Amount of change in the buccolingual inclination of the second molar

The mean amount of change in the buccolingual inclination of the second molar in the MIA group was -0.13 ± 0.66^o^ and in the CA group was 4.56 ± 0.5^o^. This showed that in the MIA group, the second molar moved lingually but minimally, whereas in the CA group, the second molar moved significantly to the buccal. The difference in the amount of change in the buccolingual inclination of the second molar between the MIA and the CA groups was found to be highly statistically significant(*p*< 0.001).

The study of Kojima et al^10^ showed that bending the spring arm produced a force tending to move the second molar in the buccal direction. In the present study the second molar also moved towards the buccal, which is corroborated by the above mentioned FEM study.

### Amount of extrusion of the second molar in the vertical plane

The mean amount of extrusion of the second molar in the MIA group was -0.03 ± 0.03 mm and in the CA group it was -0.4 ± 0.17 mm. This showed that significant extrusion of the mandibular second molar occurred in the CA group, whereas in the MIA group, the second molar extruded very low. This is corroborated by several authors^6^ ^-^ ^11^ who have stated that extrusion is an unfortunate complication of the conventional uprighting springs. However other authors^15^ ^,^ ^16^ have shown how extrusion is minimal with mini-implant assisted molar uprighting. The difference in the amount of molar extrusion between the MIA and CA groups was found to be statistically significant (*p*< 0.05). The growth pattern of the patient may have been a confounding factor as far as extrusion of molars was concerned. However there is no evidence in literature thus far to explain to what degree the same could have affected the results.

### Comparison of the amount and type of uprighting movement of the second molar over a period of four months in the MIA and CA groups

The mean amount of molar uprighting which took place by root movement was 0.64 mm and that which occurred by distal crown tipping was 0.14 mm. This result suggested that a more significant amount of molar uprighting occurred by mesial root movement in the MIA group. The mean difference of the two types of molar uprighting movements was 0.5 mm, which was statistically significant (*p*< 0.05).

The mean amount of molar uprighting which took place by root movement was 0.24 mm and that which occurred by distal crown tipping was 1.03 mm. This result suggested that a more significant amount of molar uprighting occurred by distal crown tipping in the CA group. The mean difference of the two types of molar uprighting movements was -0.79 mm, which was statistically significant (*p*< 0.05).

In the CA group, the type of uprighting is in accordance with Sawicka et al,^3^ who stated that molar uprighting with the conventional uprighting spring results in distal crown tipping and opening of the prosthetic space as a result of the counterclockwise moment created on the molar, whereas in the MIA group, a larger amount of molar uprighting occurred by mesial root movement, which is in accordance with Derton et al^14^ study, where the third molar moved mesially as it was uprighted. 

## CONCLUSIONS

The following were the conclusions drawn from the present study:

1. Significant amount of molar uprighting can be attained by both conventional helical uprighting spring (CA group) and mini-implant assisted molar uprighting (MIA group) methods, and is not affected by the type of anchorage used.
2. Mini-implant assisted molar uprighting (MIA group) was more effective in preventing the buccal movement of anchorage teeth and changes in the buccolingual inclination of the second molar, when compared to the conventional helical uprighting spring (CA group).
3. Mini-implant assisted molar uprighting (MIA)was more effective in preventing extrusion of the second molar in the vertical plane as compared to the conventional helical uprighting spring (CA group).
4. Molar uprighting in the conventional anchorage group (CA) occurred primarily by distal crown tipping whereas in the mini-implant anchorage group (MIA), it occurred primarily by mesial root movement.
